# Imaging to guide ICD therapy: beware what lies beneath

**DOI:** 10.1007/s12471-014-0593-1

**Published:** 2014-09-04

**Authors:** M. T. Rijnierse, P. Knaapen

**Affiliations:** 1 Departments of Cardiology and Institute for Cardiovascular Research (IcaR-VU), VU University Medical Center, Amsterdam, the Netherlands; 2 Department of Cardiology, VU University Medical Center, De Boelelaan 1117, PO Box 7057, 1081 HV Amsterdam, the Netherlands

The introduction of the implantable cardioverter-defibrillator (ICD) for primary prevention of sudden cardiac death has led to an important reduction in mortality in patients with impaired left ventricular ejection fraction (LVEF) [1, 2]. Consequently, current clinical guidelines recommend ICD implantation for primary prevention in patients with an LVEF below 30–40 % [3]. Although the landmark trials upon which these guidelines are based have utilised various imaging modalities to assess LVEF, two-dimensional (2D) echocardiography has been most frequently used to enrol patients. Furthermore, in clinical practice 2D echocardiography is still most commonly performed for this purpose, as it is inexpensive and readily available. In recent years, however, cardiovascular magnetic resonance imaging (CMR) has emerged as the preferred modality to quantify left ventricular volumes and LVEF due to its high reproducibility and accuracy. As such, CMR is often referred to as the gold standard for LVEF assessment [4]. The question arises, whether these imaging modalities are interchangeable for evaluating the eligibility of ICD therapy. This issue becomes particularly relevant in patients with borderline LVEF values (30–40 %) for primary prevention of sudden cardiac death when LVEF is virtually the only parameter to guide such a decision.

**Fig. 1:**
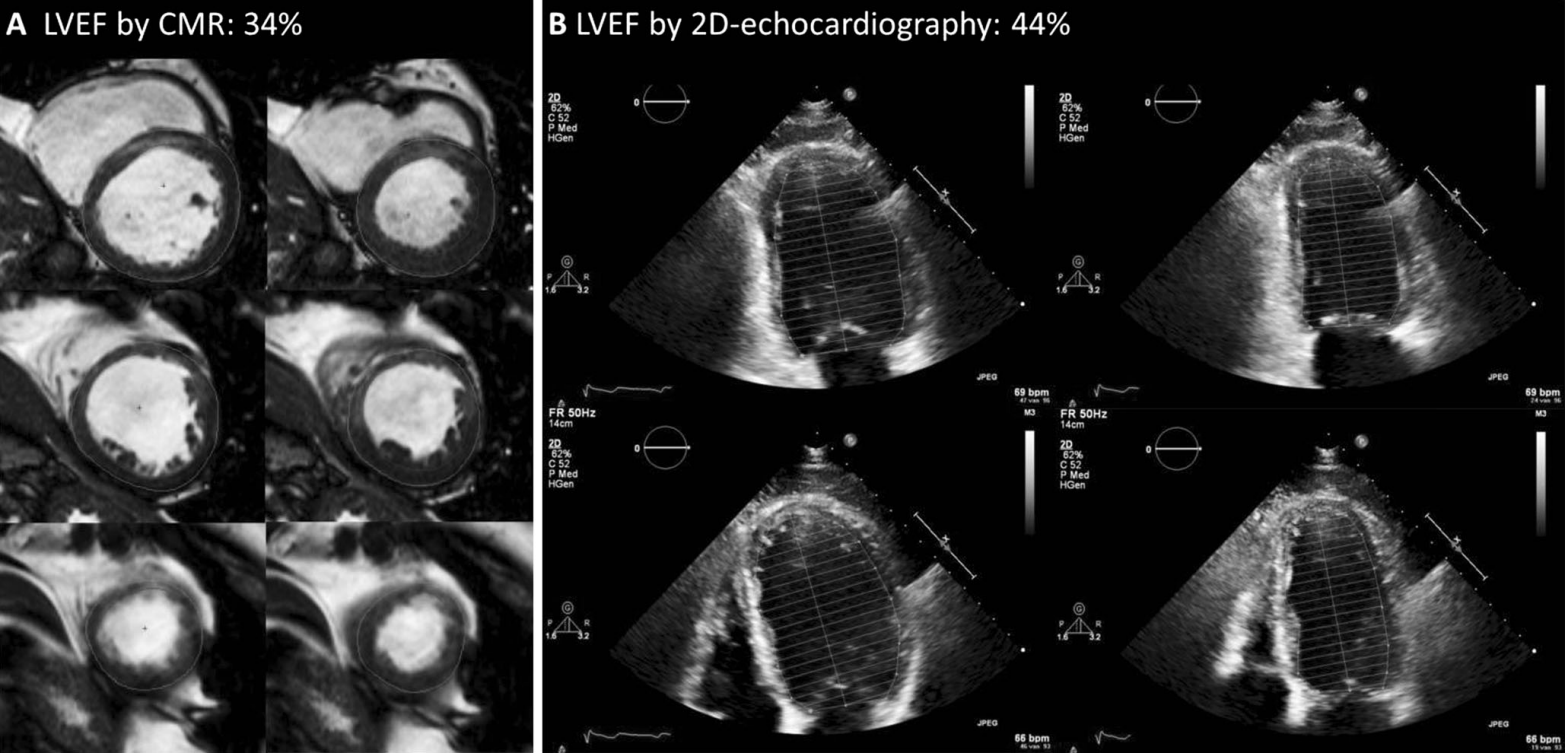
A 67-year-old patient with ischaemic cardiomyopathy who was screened for ICD implantation for primary prevention of sudden cardiac death. No arrhythmias were documented and the patient did not show signs of heart failure. Both 2D echocardiography and CMR were performed, which yielded a difference in LVEF of 10 % (44 vs. 34 %, respectively). Based on echocardiographic analysis, ICD implantation should not have been performed, yet CMR drove the decision to implant the device. To date, 2 years after implantation, no ventricular arrhythmic events have occurred

The study performed by De Haan et al. published in the current issue of the Netherlands Heart Journal included 152 patients who were referred for primary prevention ICD implantation [5]. All patients underwent LVEF assessment by 2D echocardiography as well as CMR within three months prior to device implantation. De Haan et al. report significantly lower values of left ventricular volumes by 2D echocardiography, which on average yielded a higher LVEF of 6.6 %. Although this percentage might sound trivial, from a clinical point of view such an apparent small difference can have great consequences (see Fig. 1). In fact, when taking the most utilised LVEF cut-off value of 35 % by 2D echocardiography to determine device eligibility, 28 % of patients were reclassified when CMR was used as a reference. Apparently, the choice of imaging has a substantial impact on the decision process of selecting appropriate patients for ICD implantation. Obviously, this raises concern and leads to the question which modality to choose. When stakes are that high for the care of our patients, knowledge on the background and characteristics of such imaging modalities becomes imperative.

The lack of uniformity between imaging modalities to assess LVEF has been well documented. Although comparative studies have shown some discrepancies, most of the data suggest that LVEF tends to be highest when utilising 2D echocardiography and subsequently gradually declines with CMR and nuclear ventriculography, respectively [6]. The quality of echocardiography heavily relies on an adequate acoustic window and bi-plane Simpson’s delineation of the endocardial contours may not be obtained in as many as 30 % of patients. Moreover, 2D echocardiography is based on geometric assumptions of the left ventricle, which poses a source of error in patients with heart failure and LV remodelling. Even with sufficient image quality, the reproducibility of 2D echocardiography has proven to be inferior to CMR [7]. Consequently, many centres nowadays consider CMR to be the imaging modality of choice to screen patients for ICD eligibility. In line with the current results of De Haan et al., Joshi and colleagues have also recently highlighted that CMR guided ICD therapy results in an approximately 25 % augmented implantation rate as compared with echocardiography [8]. Although long-term follow-up and comparison of the event rate of echo vs. CMR driven ICD therapy is lacking, one could logically hypothesise that appropriate ICD therapy for ventricular arrhythmic events will decrease for CMR based implantation when identical LVEF cut-off values are applied. As a consequence, health care costs will steadily rise whereas the relative benefit for our patients diminishes. As correctly pointed out by De Haan et al., the LVEF threshold to guide ICD therapy when utilising CMR should be redefined and will likely need to be adjusted to lower levels.

So what should our practice be in the meantime? As current clinical guidelines for ICD implantation are predominantly based on studies using 2D echocardiography, it could be argued that this type of imaging should be preferred to practice evidence-based medicine. Indeed, the ACC/AHA/ESC 2006 guidelines for ICD implantation actually recommend echocardiography for the assessment of left ventricular function [3]. It is of interest to note that the more recent ACC/AHA/HRS 2008 guidelines have nuanced this recommendation and no longer specify an imaging modality to assess LVEF, as long as it is ‘… *the most clinically accurate and appropriate in their institution*’ [9]. The current data from De Haan et al. remind us that different imaging modalities should certainly not be considered interchangeable and we should be aware what lies beneath the obtained results of a certain requested test to guide the clinical course of our patients. Even though it is clear that CMR holds great potential to act as gatekeeper for ICD therapy, caution on its interpretation is warranted. Future studies should focus on the clinical consequences of CMR based evaluation of ICD eligibility for primary prevention of sudden cardiac death.
